# Field Performance of a Rapid Test to Detect Progressive, Regressive, and Abortive Feline Leukemia Virus Infections in Domestic Cats in Australia and Germany

**DOI:** 10.3390/v15020491

**Published:** 2023-02-10

**Authors:** Mark E. Westman, Juliana Giselbrecht, Jacqueline M. Norris, Richard Malik, Jennifer Green, Elle Burton-Bradley, Ashley Cheang, Theres Meili, Marina L. Meli, Katrin Hartmann, Regina Hofmann-Lehmann

**Affiliations:** 1 Sydney School of Veterinary Science, The University of Sydney, Sydney, NSW 2006, Australia; 2 Clinic of Small Animal Medicine, Centre for Clinical Veterinary Medicine LMU Munich, Veterinaerstrasse 13, 80539 Munich, Germany; 3 Clinical Laboratory, Department of Clinical Diagnostics and Services, and Center for Clinical Studies, Vetsuisse Faculty, The University of Zurich, CH-8057 Zurich, Switzerland; 4 The Sydney Institute for Infectious Diseases, The University of Sydney, Sydney, NSW 2006, Australia; 5 Centre for Veterinary Education, The University of Sydney, Sydney, NSW 2006, Australia; 6 School of Veterinary and Animal Science, Charles Sturt University, Wagga Wagga, NSW 2678, Australia

**Keywords:** antibodies, FeLV, FIV, infection, humoral immunity, v-RetroFel^®^, vaccination, veterinary science

## Abstract

Different feline leukemia virus (FeLV) infection outcomes are possible in cats following natural exposure, such as progressive infections (persistent viremia), regressive infections (transient or no viremia followed by proviral persistence) and abortive infections (presence of only antibodies). Laboratory-based testing is currently required for categorization of infection outcomes in cats. The aim of this study was to evaluate the field performance of a novel, rapid, combination point-of-care (PoC) test kit commercially available in Europe (v-RetroFel^®^Ag/Ab; 2020–2021 version) to determine different FeLV infection outcomes by concurrent detection of FeLV antigen (p27) and antibodies against FeLV transmembrane envelope protein (p15E). A secondary aim was to evaluate the performance of the same test kit (v-RetroFel^®^FIV) to determine positive/negative feline immunodeficiency virus (FIV) infection status by the detection of antibodies to FIV capsid protein (p24) and transmembrane glycoprotein (gp40). Two cohorts of domestic cats were recruited and tested with v-RetroFel^®^ using plasma or serum, including cats in Australia (*n* = 200) and cats in Germany (*n* = 170). Results from p27 antigen PoC testing, proviral DNA PCR, and neutralizing antibody testing or testing for antibodies against non-glycosylated surface unit envelope protein (p45) were used to assign cats to groups according to different FeLV infection outcomes. Testing with a laboratory-based FeLV p15E antibody ELISA was also performed for comparison. In the first cohort, v-RetroFel^®^Ag/Ab correctly identified 89% (109/122) FeLV-unexposed cats and 91% (21/23) progressive infections, but no regressive (0/23) or abortive (0/32) infections. In the second cohort, v-RetroFel^®^Ag/Ab correctly identified 94% (148/158) FeLV-unexposed cats and 100% (4/4) progressive infections, but no regressive (0/2) and only 17% (1/6) abortive infections. There was test agreement between v-RetroFel^®^Ab and the p15E laboratory ELISA in 58.9% of samples. As a secondary outcome of this study, the sensitivity and specificity of v-RetroFel^®^FIV testing in cohort 1 were 94.7% (18/19) and 98.3% (178/181), and in cohort 2, 30.0% (3/10) and 100.0% (160/160), respectively. Prior history of FIV vaccination did not produce any false-positive FIV results. In conclusion, v-RetroFel^®^Ag/Ab (2020–2021 version) was unable to accurately determine different FeLV infection outcomes in the field. Improvements of the test prior to application to field samples are required.

## 1. Introduction

Feline leukemia virus (FeLV) is a *Gammaretrovirus* that infects domestic and non-domestic felids worldwide [1,2,3,4,5]. Both exogenous and endogenous forms of FeLV have been identified, with recombination and mutation events giving rise to different FeLV subgroups [1,6,7,8,9,10,11,12,13]. Exogenous FeLV-A is the subgroup almost exclusively transmitted horizontally between cats [14].

Exposure to exogenous FeLV-A produces a spectrum of possible outcomes in cats, depending on the challenge dose, virus virulence, infection pressure (e.g., single exposure vs. extended contact), cat age, and host immunity factors [15,16,17,18,19,20,21]. Terminology used to describe different categories of FeLV infection has developed over time with the advent of molecular testing [15,22,23,24]. Currently, both European and North American guidelines on the prevention, diagnosis, and management of FeLV have adopted the nomenclature of progressive, regressive, and abortive infections [25,26].

Progressively infected cats are persistently viremic, with a primary viremia involving local oropharyngeal lymphoid tissue (duration 1–12 weeks) followed by a secondary viremia caused by infection of the bone marrow (2–16 weeks and beyond) [15,26,27]. Progressively infected cats have a poor prognosis, and can eventually develop disorders of hematopoiesis, immune suppression, and neoplasia, resulting in a prognosis for survival of only three years for up to 80–90% of infected cats [25,27,28,29,30].

In regressively infected cats, a primary viremia usually (but not always) occurs before a sufficient host immune response is mounted to clear the viremia [15,26,27], but lifelong infection in the form of proviral DNA integration results [15,26,29]. The prognosis for regressive infections varies, with some studies reporting an association with lymphomagenesis; additionally, regressive infections can be reactivated [31,32,33,34,35]. 

Cats with abortive infections are never viremic and resist proviral integration due to a timely and robust immune response, and carry the same long-term prognosis as FeLV-uninfected cats [15,21,26].

A gamut of testing is required to classify the type of FeLV infection following exposure. The mainstay of FeLV screening is detection of viral capsid protein (p27) with rapid point-of-care (PoC) test kits (antigen testing) [15,26,36]. Other available FeLV testing options, depending on the country, include PCR testing to detect proviral DNA in blood or bone marrow, immunofluorescent antibody testing to detect cell-associated p27-antigen, virus isolation (VI) to detect viable virus presence in body fluids, and reverse-transcriptase (RT)-PCR testing to detect viral RNA in blood or saliva [15,21,37,38,39,40,41,42]. Since progressive and regressive infections can be indistinguishable very early in the course of infection (both being p27-positive, PCR-positive and RT-PCR-positive during the viremic phase), repeat p27-antigen testing can be required to differentiate progressive (persistently p27-positive) and regressive infections (transiently p27-positive) [15,29,43,44,45].

Antibody testing can be useful for identifying FeLV exposure and assigning a category of infection [15,16,42,44,46]. Regressive and abortive infections, but not progressive infections, usually have a detectable neutralizing antibody (NAb) response [15,20,43,47]. All FeLV-infected cats, irrespective of infection outcome (i.e., progressive, regressive, or abortive), are assumed to develop antibodies against the FeLV transmembrane protein (p15E) [48,49]. Progressive infections, however, have been reported to have weaker immunoblot and p15E enzyme-linked immunosorbent assay (ELISA) reactions than regressive and abortive infections [50]. Based on results from a laboratory-based ELISA to detect anti-p15E antibodies [49], the first commercially available FeLV antibody PoC test kit (v-RetroFel^®^, Scil Animal Care Company, Viernheim, Germany) was launched in Europe in April 2018, with the manufacturer claiming test results can discriminate between different infection types.

Regressively infected cats have been demonstrated to transmit FeLV infection to FeLV-naive cats via blood transfusion [51]. Consequently, FeLV PCR testing of all donor cats prior to blood transfusion is recommended to identify regressive infections and facilitate the removal of these cats from blood donor programs [52]. Since commercial PCR testing usually involves a 1–3 day turnaround for results, when a rapid blood transfusion is required, this approach is usually not possible. Therefore, having the ability to identify regressively infected cats in an acute emergency setting quickly and easily by PoC testing prior to blood transfusion would be advantageous [52]. Similarly, rapid identification of regressively infected cats in multi-cat household situations by PoC testing would be useful [15,21]. Accurate and rapid identification of regressive and abortive infections would also be beneficial for veterinarians trying to make informed risk–benefit assessments regarding FeLV vaccination for cats in their local area, since it provides a more accurate estimate of the true FeLV prevalence and therefore, in general, of the risk of FeLV exposure [53]. FeLV PoC antigen testing is able to differentiate infected from vaccinated animals (DIVA) [26], and laboratory-based p15E antibody testing has shown promise as a potential DIVA test [48,49].

The primary aim of this study was to assess the field performance of v-RetroFel^®^Ag/Ab (2020–2021 version) to detect different FeLV infection outcomes using samples collected in Australia and Germany. A secondary aim was to evaluate the performance of v-RetroFel^®^FIV (a third strip present within the same v-RetroFel^®^ combination test kit) to determine feline immunodeficiency virus (FIV) infection status in the same population of cats.

## 2. Materials and Methods

### 2.1. Australian Samples (Cohort 1; n = 200)

Residual plasma samples from previous studies were utilized for FeLV antibody testing [47,54]. Samples included client-owned cats and cats residing in two rescue facilities [47]. Blood was collected by jugular or cephalic venipuncture following application of a local anesthetic cream and was then immediately aliquoted into multiple ethylenediamine tetra-acetic acid (EDTA) tubes. An EDTA tube was centrifuged for 3 min at 12,000× *g*, and harvested plasma was aliquoted into two plain tubes using a sterile pipette and stored at −80 °C until use.

In total, 93/200 (46.5%) cats were FeLV-unvaccinated and 107/200 (53.5%) cats were FeLV-vaccinated: 38 cats (19%) had been vaccinated against FeLV with a monovalent inactivated whole-virus (IWV) vaccine (Fel-O-Vax^®^ Lv-K, Boehringer Ingelheim Animal Health, Fort Dodge, IA, USA); 50 cats (25%) had been vaccinated with a polyvalent vaccine, which included inactivated whole FeLV antigen (Fel-O-Vax^®^ 5, Boehringer Ingelheim Animal Health); and 19 cats (9.5%) were vaccinated with a monovalent FeLV subunit p45 vaccine (Leucogen^®^, Virbac Animal Health, Bendigo, Victoria, Australia). Additionally, 29 cats (14.5%) had been vaccinated against FIV with a dual-subtype IWV vaccine (Fel-O-Vax^®^ FIV, Boehringer Ingelheim Animal Health). One cat had been vaccinated against both FeLV (Fel-O-Vax^®^ 5) and FIV.

Commercially available FeLV PoC testing (SNAP Combo^®^, IDEXX Laboratories, Westbrook, ME, USA; Witness^®^, Zoetis Animal Health, Lyon, France; or Anigen Rapid^®^, BioNote, Gyeonggi-do, Republic of Korea) was performed with fresh EDTA anticoagulated whole blood to detect p27-antigenemia. SNAP Combo^®^ has published sensitivity and specificity under the Australian conditions of 100% and 94%, while Witness^®^ and Anigen Rapid^®^ both have published sensitivity and specificity values of 91% and 98% under Australian conditions [55]. All p27-positive results were confirmed either by testing with a second FeLV PoC test (from a different manufacturer), or, following transfer of plasma, stored at −80 °C on dry ice and transported to Clinical Laboratory, Vetsuisse Faculty, the University of Zurich, testing with a laboratory-based p27 antigen sandwich ELISA [56]. Some results were confirmed by both methods. Residual plasma from regressive and abortive infections was also used for p27 antigen laboratory ELISA testing when available.

Plasma stored at −80 °C and transferred on dry ice to Clinical Laboratory, Zurich was also tested for anti-p15E antibodies using a laboratory ELISA as described [49]. Cloned and purified whole p15E subunit of FeLV-A (GenBank accession no. AAA93093.1), without the membrane-spanning helix part of the viral envelope protein, was used as the ELISA capture antigen [49]. Relative optical density (ROD) values were determined using the formula ROD = [(Sample OD − Negative control OD)/(Positive control OD − Negative control OD)]. Samples with ROD that tested > 16.3% (ROD value 0.163) compared to the positive control (pooled serum sample from cats experimentally infected with FeLV-A/Glasgow-1) were considered antibody-positive, as was determined for cats in a previous Swiss field study [49].

FeLV real-time (q)PCR testing to detect proviral DNA was performed on EDTA anticoagulated whole blood samples in duplicate at Veterinary Pathology Diagnostic Services (VPDS), Sydney School of Veterinary Science (SSVS), the University of Sydney, as described [57,58].

Plasma stored at −80 °C was transferred on dry ice to Veterinary Diagnostic Services (VDS), the University of Glasgow, for FeLV NAb testing, as described [47,59]. Twofold serial dilutions of plasma samples (1/4, 1/8, 1/16, and 1/32) were tested, and any dilution that reduced the focus count of FeLV by 75% compared with the virus control was considered a positive result [47].

Table 1 summarizes the testing approach used to classify cats in Australia as progressively infected (*n* = 23), regressively infected (*n* = 23), abortively infected (*n* = 32), or FeLV-unexposed (*n* = 122).

The accuracy of v-RetroFel^®^FIV to determine FIV infection status was also evaluated. In total, samples from 19 FIV-infected cats (including 2 annually FIV-vaccinated cats) and 181 FIV-uninfected cats (including 27 annually FIV-vaccinated cats) were tested. The FIV status of all 200 samples had been previously determined with fresh EDTA anticoagulated whole blood using FIV PoC test kits from the same three manufacturers as the FeLV PoC test kits (i.e., SNAP Combo^®^, Witness^®^, or Anigen Rapid^®^). The sensitivity and specificity of each test kit under Australian conditions were reported (100% and 64% for SNAP Combo^®^, 100% and 98% for Witness^®^, and 100% and 100% for Anigen Rapid^®^) [60]. FIV-positive results were confirmed by testing with the other two FIV PoC tests, and in most FIV-positive cases (15/19), FIV PCR testing to detect viral RNA and proviral DNA was conducted (FIV RealPCR^®^, IDEXX Laboratories, East Brisbane, QLD, Australia). The sensitivity and specificity of FIV RealPCR^®^ testing under Australian conditions were reported to be 92% and 99% [60]. Virus isolation was used to confirm FIV infection in the two FIV-vaccinated cats at the University of Florida and the University of Glasgow [60,61].

Plasma stored at −80 °C was thawed for testing with v-RetroFel^®^PoC kits at SSVS. Two observers (Jennifer Green, and E.B-B. or A.C.), blinded to the FeLV and FIV infection status of all samples, performed v-RetroFel^®^ PoC testing in accordance with manufacturer’s instructions. Both observers were in agreement for all test results.

### 2.2. German Samples (Cohort 2; n = 170)

Serum and EDTA anticoagulated whole blood samples from 170 cats in Germany were collected prospectively. Samples originated from cats that presented at the Clinic of Small Animal Medicine of the Centre for Clinical Veterinary Medicine LMU Munich and had blood drawn for various reasons, as well as shelter cats with unknown FeLV and FIV status. Both cats with a history of illness and healthy cats were tested. Of the 170 cats, 11 were vaccinated against FeLV. Five cats were vaccinated with a recombinant canarypox virus (vCP97) vaccine (Purevax^®^ FeLV, Boehringer Ingelheim Vetmedica GmbH, Rohrdorf, Germany), four cats with a monovalent FeLV subunit vaccine (Leucogen^®^, Virbac Animal Health, Carros, France), and in two cats, the FeLV vaccine administered was unknown. None of the cats were vaccinated against FIV.

Samples were stored at −80 °C for a maximum of 24 months before being sent on dry ice to Clinical Laboratory, Vetsuisse Faculty, the University of Zurich.

Samples were tested for the presence of free FeLV p27 antigen in serum by sandwich ELISA, as described previously [56]. All samples were tested in duplicate and the absorbances were read using a microplate reader (Synergy H1, Biotek, VT, USA).

To confirm positive p27 antigen results, blood and saliva samples from all p27 antigen-positive cats (*n* = 4) were tested for viral RNA. A published RT-qPCR assay [37,58] was used to detect FeLV viral RNA, with each sample being tested once. Positive and negative controls were run in parallel with each RT-qPCR. All negative samples were diluted 1:5 and 1:10 in a neutral buffer at pH 7.4 (0.15 M sodium chloride, 1 mM EDTA, 0.05 MTris-base, 0.1% BSA, 0.1% Tween 20) to make possible inhibition unlikely.

For FeLV proviral DNA testing [58], total nucleic acids [49] were extracted from 100 µL EDTA anticoagulated whole blood using the MagNa Pure 96 instrument (Roche Diagnostics AG, Rotkreuz, Switzerland) and the Viral NA SV Kit (Roche Diagnostics AG, Rotkreuz, Switzerland) with a 100 µL elution buffer according to the manufacturer’s instructions. For all samples, the viral NA plasma external lysis SV 4.0 protocol (Roche Diagnostics AG) was applied, with each sample being tested once, and negative controls of phosphate-buffered saline (PBS) were run in parallel with each batch of samples to monitor for cross-contamination.

The proviral DNA copy number was amplified and quantified using 5 μL of TNA and 20 µL of DNA quantitative PCR Mastermix (Eurogentec, Seraing, Belgium) containing 480 nM primers (exoFeLV-U3F2, exoFeLV-U3R3) and a 160 nM probe (exoFeLV-U3p). All oligonucleotides were synthetized by Microsynth AG (Balgach, Switzerland). The temperature profile consisted of 2 min at 50 °C, denaturation for 10 min at 95 °C, followed by 45 cycles of 95 °C for 15 s and 60 °C for 1 min. The FeLV proviral copy numbers in the single samples were determined by co-amplifying 10-fold serial dilutions of a DNA standard template, as described previously [6]. All samples that tested positive in the p27 antigen ELISA were diluted 1:5 and 1:10 in the neutral buffer to avoid a false negative result in the provirus qPCR due to possible inhibition. To verify the quantity and quality of viral load, quantitative PCR for feline albumin was performed on all 170 TNA samples [62].

Serum samples were analyzed for the presence of antibodies against FeLV surface unit (SU) non-glycosylated protein (p45), FeLV whole virus (FL-74), and FeLV p15E using indirect ELISAs, as described previously [16,44,49]. Anti-SU and anti-whole virus antibody concentrations > 25% (ROD value 0.250) [63] and anti-p15E antibody concentrations > 16.3% (ROD value 0.163) [49], compared to the positive control (pooled serum sample from cats experimentally infected with FeLV-A/Glasgow-1), were defined as antibody-positive.

Table 2 summarizes the testing approach used to classify cats in Germany as progressively infected (*n* = 4), regressively infected (*n* = 2), or abortively infected (*n* = 6), or FeLV-unexposed (*n* = 158).

In addition, all samples were tested for the presence of FIV antibodies by Western blotting (WB) to determine FIV status. The WB was performed as described [64,65,66,67], and samples were considered WB-positive (i.e., FIV-infected) if two bands with a molecular weight of 15,000 (p15) and 24,000 (p24) Daltons, respectively, were identifiable on the blotting strip [64]. If both bands were absent, the sample was classified as WB-negative (i.e., FIV-uninfected). Samples that had only one band, either p15 or p24, were classified as FIV-negative at the time of sampling.

Serum centrifuged directly after blood sampling was used to perform v-RetroFel^®^ PoC testing. Two observers (Juliana Giselbrecht and a second person) performed and interpreted the tests at the Small Animal Clinic, LMU Munich. The tests were performed according to the manufacturer’s instructions. At the time that v-RetroFel^®^ PoC testing was performed, the results of the FeLV and FIV laboratory results were unknown. Both observers were in agreement for all test results.

### 2.3. Evaluation of v-RetroFel^®^ Test Results

The v-RetroFel^®^ PoC test consists of three separate test strips designed to detect (i) FeLV p27 antigen, (ii) antibodies to FeLV transmembrane protein (p15E), and (iii) antibodies to FIV capsid protein (p24) and transmembrane glycoprotein (gp40).

For the current study, when presenting results from v-RetroFel^®^ PoC testing, the following abbreviations will be used hereafter: (i) v-RetroFel^®^Ag for PoC FeLV p27 antigen results; (ii) v-RetroFel^®^Ab for PoC FeLV p15E antibody results; and (iii) v-RetroFel^®^FIV for PoC FIV antibody results. For combined p27 antigen/p15E antibody results, v-RetroFel^®^Ag/Ab will be used.

For v-RetroFel^®^Ag/Ab testing, the manufacturer claims that:

- p27-positive/p15E antibody-positive results indicate progressive or early regressive FeLV infections;
- p27-negative/p15E antibody-positive results indicate regressive or abortive FeLV infections;
- p27-negative/p15E antibody-negative results indicate no exposure to FeLV;
- p27-positive/p15E antibody-negative results are unlikely to be observed (but would also be considered indicative of progressive infections).

For v-RetroFel^®^FIV testing, results are reported as antibody-positive (FIV-infected) or antibody-negative (FIV-uninfected).

Test agreement between v-RetroFel^®^Ab and p15E laboratory ELISA results was calculated using both negative and positive test results. Test outcomes for v-RetroFel^®^ were compared between groups with each study population by Fisher’s exact testing, and results from p15E laboratory ELISA testing were compared between groups by Mann–Whitney *U*-testing since data were not normally distributed. Ages were compared by two-tailed *t*-testing. For all analyses, a *p* value < 0.05 was considered significant. Sensitivity and specificity for FIV testing with 95% confidence intervals (CI) were calculated using Microsoft Excel^®^.

## 3. Results

### 3.1. Australian Samples (n = 200)

A summary of the results is shown in Table 3.

#### 3.1.1. Results of v-RetroFel^®^Ag/Ab Testing

Overall, using v-RetroFel^®^Ag/Ab, 122/200 (61%) of cases werein agreement with this study’s definitions of FeLV infection status.

Testing with v-RetroFel^®^Ag/Ab correctly identified 91% (21/23) progressive infections (13 cats tested antigen-positive/antibody-positive and 8 cats tested antigen-positive/antibody-negative) but did not correctly identify any regressive (0/23) or abortive (0/32) infections (all 55 cats incorrectly tested antibody-negative). Two progressively infected cats tested falsely antigen-negative (and antibody-negative) with v-RetroFel^®^Ag/Ab. One FeLV-unexposed cat tested falsely antigen-positive (and antibody-negative) with v-RetroFel^®^Ag/Ab.

Testing with v-RetroFel^®^Ag/Ab correctly identified 89% (109/122) FeLV-unexposed cats (antigen-negative and antibody-negative). In addition to the one cat that tested falsely antigen-positive (and antibody-negative), 12 cats tested antigen-negative/antibody positive (all 12 had been vaccinated against FeLV). In the 122 FeLV-unexposed cats, antibody-positive results with v-RetroFel^®^Ab were more likely to occur in younger cats than older cats (1.3 years mean age for antibody-positive results vs. 5.1 years for antibody-negative results; *p* = 0.0003; two-tailed *t*-test). Males were less likely to test antibody-positive than females (3/67 males vs. 9/55 females; *p* = 0.035; Fisher’s exact test). FeLV-unexposed cats that had been vaccinated against FeLV (*n* = 82) were more likely to test antibody-positive with v-RetroFel^®^Ab than unvaccinated FeLV-unexposed cats (*n* = 40) (12/82 vs. 0/40; *p* = 0.02; Fisher’s exact test). None of the three FeLV vaccines was more likely than the others to produce antibody-positive results with v-RetroFel^®^Ab testing in the 82 FeLV-vaccinated/FeLV-unexposed cats (Fel-O-Vax^®^ Lv-K—5/27, Fel-O-Vax^®^ 5—4/36, and Leucogen^®^—3/19; *p* > 0.48; Fisher’s exact testing).

The sensitivity and specificity of v-RetroFel^®^Ag for p27 antigen were 91.3% (21/23) and 99.4% (176/177) respectively. For anti-p15E antibodies, the sensitivity and specificity of v-RetroFel^®^Ab (based on the assumption that all progressively, regressively, and abortively infected cats produce antibodies against p15E) were 16.7% (13/78) and 90.2% (110/122) respectively.

#### 3.1.2. Results of p15E Laboratory ELISA Testing

**Figure 1:**
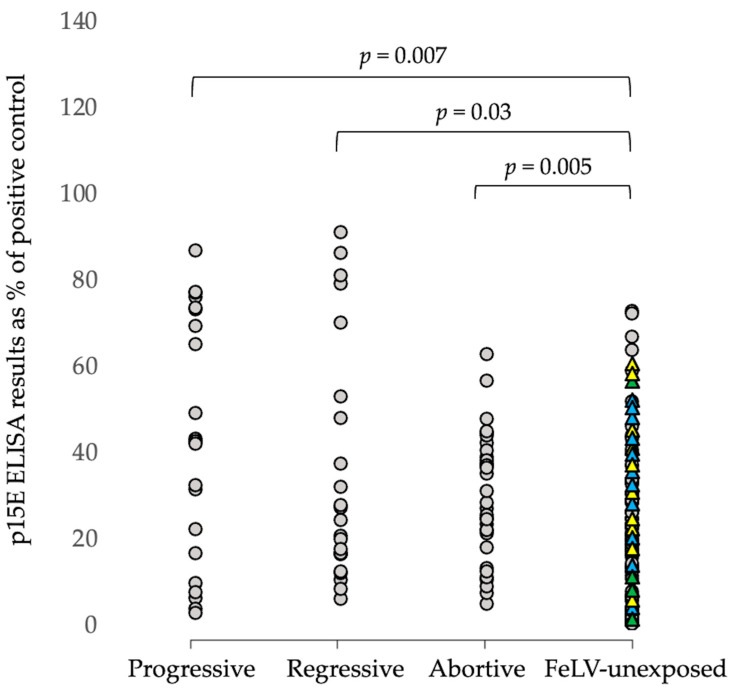
Results from p15E laboratory ELISA testing in cats in Australia (cohort 1, *n* = 200). Feline leukemia virus (FeLV)-unexposed cats had significantly lower antibody levels than progressive, regressive, and abortive infections, although there was a substantial overlap in results. The triangles represent the FeLV-vaccinated unexposed cats. The yellow triangles represent cats that had been vaccinated with Fel-O-Vax^®^ Lv-K; the blue triangles, Fel-O-Vax^®^ 5; and the green triangles, Leucogen^®^. Higher anti-p15E antibody levels were found in cats vaccinated with Fel-O-Vax^®^ Lv-K or Fel-O-Vax^®^ 5 than cats vaccinated with Leucogen^®^.

Testing with the p15E laboratory ELISA detected antibodies in 70% (16/23) progressive infections, 70% (16/23) regressive infections, and 78% (23/32) abortive infections. There were no differences in p15E laboratory ELISA levels between progressive, regressive, and abortive infections (*p* < 0.35; Mann–Whitney *U*-testing). More than half of FeLV-unexposed cats, however, also tested p15E laboratory ELISA-positive (70/122; 57%). In the 122 FeLV-unexposed cats, p15E laboratory ELISA-positive results were more likely to occur in older cats than younger cats (6.9 years mean age for antibody-positive results vs. 1.9 years for antibody-negative results; *p* < 0.00001; two-tailed *t*-test). Males were more likely to test antibody-positive than females (46/67 males vs. 24/55 females; *p* = 0.0062; Fisher’s exact test). Overall, FeLV-unexposed cats had lower p15E laboratory ELISA levels than progressively, regressively, and abortively infected cats (*p* = 0.007, *p* = 0.03, and *p* = 0.005, respectively; Mann–Whitney *U*-testing), although there was a substantial overlap in results (Figure 1).

Both FeLV-vaccinated/FeLV-unexposed cats (32/82) and unvaccinated FeLV-unexposed cats (38/40) tested p15E laboratory ELISA-positive. Surprisingly, FeLV-unvaccinated unexposed cats had higher anti-p15E antibody levels than FeLV-vaccinated unexposed cats (ROD 0.310 vs. 0.155; *p <* 0.00001; Mann–Whitney *U*-test). Of the FeLV-vaccinated unexposed cats, Fel-O-Vax^®^ Lv-K (13/27) and Fel-O-Vax^®^ 5 (18/36) produced more p15E laboratory ELISA-positive results than Leucogen^®^ (1/19; *p* < 0.01 for both, Fisher’s exact testing), and higher p15E antibody levels were observed in cats vaccinated with Fel-O-Vax^®^ Lv-K or Fel-O-Vax^®^ 5 (*p* < 0.002 for both; Mann–Whitney *U*-testing).

There was test agreement between v-RetroFel^®^Ab and p15E laboratory ELISA testing in only 78/200 (39%) of samples.

#### 3.1.3. Results of v-RetroFel^®^FIV Testing

Results are summarized in Table 4. The sensitivity and specificity of FIV testing were 94.7% (18/19; 95% CI 84.7 to 100) and 98.3% (178/181; 95% CI 96.5 to 100), respectively. Prior history of FIV vaccination did not impact the results, with all 27 FIV-vaccinated/FIV-uninfected cats testing FIV-negative, while both FIV-vaccinated/FIV-infected cats tested FIV-positive.

### 3.2. German Samples (n = 170)

A summary of the results is shown in Table 5.

#### 3.2.1. Results of v-RetroFel^®^Ag/Ab Testing

Overall, using v-RetroFel^®^Ag/Ab, 149/170 (88%) of cases were in agreement with this study’s definitions of FeLV infection status.

V-RetroFel^®^Ag/Ab correctly identified 100% (4/4) progressive infections (all 4 cats tested antigen-positive/antibody-negative) but did not correctly identify any regressive infections (0/2) and only correctly identified 17% (1/6) of the abortive infections.

Testing with v-RetroFel^®^Ag/Ab correctly identified 94% (148/158) FeLV-unexposed cats. No cat tested falsely antigen-positive (and antibody-negative). There was no sex effect on antibody-positive results (4/70 males vs. 6/88 females; *p* = 1.0; Fisher’s exact test). Ten FeLV-unexposed cats tested antigen-negative/antibody-positive (two cats had been vaccinated against FeLV, one cat with Purevax^®^ FeLV and one cat with Leucogen^®^).

The sensitivity and specificity of v-RetroFel^®^Ag for p27 antigen were 100% (4/4) and 100% (166/166) respectively. For anti-p15E antibodies, the sensitivity and specificity of v-RetroFel^®^Ab (based on the assumption that all progressively, regressively, and abortively infected cats produce antibodies against p15E) were 8.3% (1/12) and 93.7% (148/158) respectively.

#### 3.2.2. Results of p15E Laboratory ELISA Testing

**Figure 2:**
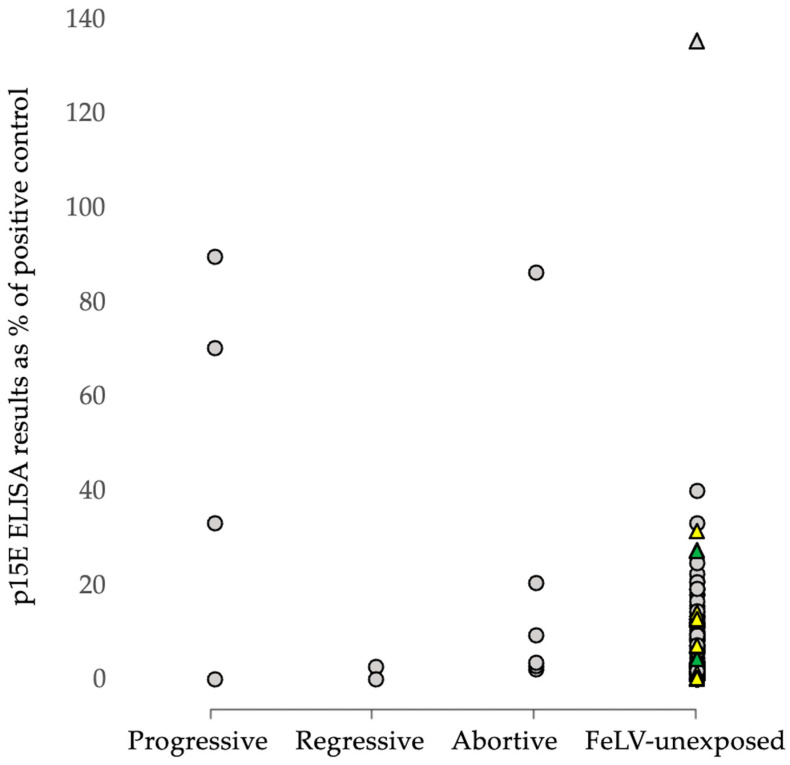
Results from p15E laboratory ELISA testing in cats in Germany (cohort 2, *n* = 170). The small numbers of cats in the progressive, regressive, and abortive categories precluded statistical analysis. The triangles represent the FeLV-vaccinated unexposed cats. The yellow triangles represent cats that had been vaccinated with Purevax^®^ FeLV; the green triangles, Leucogen^®^; and the grey triangles, an unknown vaccine manufacturer. The small numbers of vaccinated cats in each group precluded statistical analysis.

Testing with the p15E laboratory ELISA detected antibodies in 75% (3/4) progressive infections, 0% (0/2) regressive infections, and 33% (2/6) abortive infections. However, of the FeLV-unexposed cats, 8% (13/158) tested antibody-positive with the p15E laboratory ELISA (Figure 2). There was no sex effect in the FeLV-unexposed group on antibody-positive results (7/70 males vs. 6/88 females; *p* = 0.56; Fisher’s exact test). FeLV-unvaccinated unexposed cats did not have significantly different anti-p15E antibody levels compared to FeLV-vaccinated unexposed cats (ROD 0.048 vs. 0.213; *p* = 0.063; Mann–Whitney *U*-test). Sample numbers were too low to investigate a possible age effect on p15E laboratory ELISA-positive results. Antibody levels between progressively, regressively, and abortively infected cats, as well as FeLV-unexposed cats, were also not compared statistically, since the numbers of progressive, regressive, and abortive infections were too low for statistical comparison.

There was agreement between v-RetroFel^®^Ab and p15E laboratory ELISA test results in 141/170 (83%) of samples.

#### 3.2.3. Results of v-RetroFel^®^FIV Testing

Results are summarized in Table 6. Sensitivity and specificity of FIV testing were 30.0% (3/10) and 100.0% (160/160), respectively. There was no history of FIV vaccination in any cat.

### 3.3. Comparing Results from Cohorts 1 and 2

Overall, correct FeLV infection status was determined with v-RetroFel^®^Ag/Ab testing in 271/370 (73%) cases. There was no difference between countries (i.e., Australia vs. Germany) in terms of v-RetroFel^®^Ag/Ab test performance for each infection category (*p* > 0.15; Fisher’s exact tests). There was test agreement between v-RetroFel^®^Ab and the p15E laboratory ELISA in overall 58.9% (218/370) of samples.

**Figure 3:**
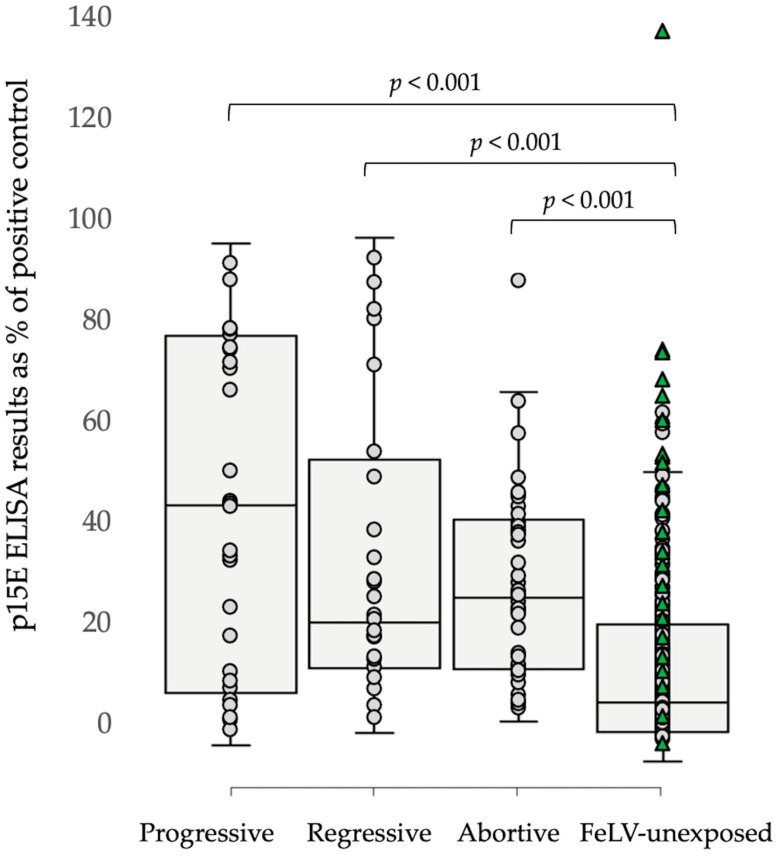
Combined results from p15E laboratory-ELISA testing in both cohorts (i.e., Australia and Germany; *n* = 370). Overall, FeLV-unexposed cats had significantly lower antibody titers than progressively (*p* = 0.00002), regressively (*p* = 0.000005), and abortively (*p* < 0.00001) infected cats. The green triangles represent FeLV-vaccinated unexposed cats.

Overall, FeLV-unexposed cats had significantly lower p15E laboratory ELISA antibody titers than progressively, regressively, and abortively infected cats (*p* = 0.00002, *p =* 0.000005, and *p* < 0.00001, Mann–Whitney *U*-tests). There was, however, a substantial overlap in antibody results between different categories (Figure 3).

The v-RetroFel^®^FIV test kit was more sensitive using samples from Australian cats (18/19 FIV-positive cats) than German cats (3/10 FIV-positive cats) (*p* = 0.0005; Fisher’s exact test), while there was no difference in test specificity between countries (178/181 vs. 160/160; *p* = 0.25; Fisher’s exact test).

## 4. Discussion

The present study evaluated a new, commercially available PoC test (v-RetroFel^®^) in naturally infected cats from Australia and Germany. The test is expected to detect different courses of FeLV infection based on the determination of p27 antigen and anti-p15E antibody status. In addition to FeLV diagnostics, the test is also marketed to detect antibodies against FIV.

The correct FeLV infection status was determined with v-RetroFel^®^Ag/Ab testing in 271/370 (73%) cases. The v-RetroFel^®^Ag/Ab PoC test identified most progressively infected cats by detecting the p27 antigen correctly (cohort 1—21/23, cohort 2—4/4). The v-RetroFel^®^Ag/Ab PoC test, however, was unable to identify regressive and abortive infections in either population of cats (cohort 1—0/55 combined, cohort 2—1/8 combined). Therefore, the version of v-RetroFel^®^Ag/Ab tested in this study (2020–2021 version) did not offer any advantages over other available PoC tests that solely detect p27 antigen, and its use cannot be recommended until improvements have been made.

The most significant form of FeLV infection is progressive infection, since these cats are the main source of infection for other, uninfected cats [68]. Progressively infected cats are more likely to develop FeLV-associated diseases, including immunodeficiency; bone marrow suppression (pancytopenias); and neoplasia, resulting in death [9,44,69,70]. In this study, both Australian and German cats were considered progressively infected if they tested positive for the presence of p27 antigen in the blood with a range of commercially available PoC kits and/or a laboratory ELISA. In addition, all progressively infected cats tested provirus PCR-positive. The v-RetroFel^®^Ag/Ab PoC test was able to identify 25/27 progressive infections within the two cohorts, but of concern were two progressive infections in the Australian cohort that would have been missed with v-RetroFel^®^Ag/Ab testing alone.

p15E is a transmembrane protein that is expressed on the surface of FeLV-infected cells, and it allows the virus to enter the host cell and inhibit lymphocyte proliferation and T-cell functions, thereby possessing immunosuppressive properties [71,72]. Antibodies directed against p15E rarely have virus-neutralizing properties [73]. Lutz and colleagues analyzed the quality and quantity of antibodies against different FeLV components in naturally infected cats and found that p15E had strong antigenicity. They observed that cats displayed elevated levels of antibodies to p15E, whether they became immune or viremic after infection [48]. In the present study, most progressively infected cats (cohort 1—16/23, cohort 2—3/4) tested positive for anti-p15E antibodies with the p15E laboratory ELISA, as did regressively infected cats (cohort 1—16/23, cohort 2—0/2) and abortively infected cats (cohort 1—25/32, cohort 2—2/6). These results support the hypothesis that most FeLV-infected cats (but not all) produce some antibodies against p15E [49].

FeLV-unexposed cats vaccinated against FeLV in cohort 1 had lower antibody titers against p15E detected by laboratory ELISA testing than unvaccinated cats, suggesting that the presence of anti-p15E antibodies indicates previous infection rather than vaccination. This finding is comparable to previous work, in which it was found that most vaccinated client-owned cats in Switzerland had p15E antibody values lower than the threshold calculated for FeLV-naive cats [49]. No difference in anti-p15E antibody levels was found between FeLV-vaccinated and FeLV-unvaccinated unexposed cats in cohort 2. In cohort 1, vaccination with an IWV FeLV vaccine (Fel-O-Vax^®^ Lv-K or Fel-O-Vax^®^ 5) produced a more reliable p15E antibody response then vaccination with the subunit vaccine (Leucogen^®^), supporting previous findings that antibody reaction depends on the vaccine administered [49]. In cohort 1, more progressively infected cats (13/23) tested positive with v-RetroFel^®^Ab than regressively and abortively infected cats (0/55). This finding, however, was not supported by results from p15E laboratory ELISA testing, with no difference in antibody levels between the types of infection found. Further studies are needed to determine to what extent the production of anti-p15E antibodies affects the different possible outcomes following FeLV exposure, how long antibodies are detectable following both FeLV vaccination and infection, and whether p15E antibody testing might predict infection.

Of concern for the p15E laboratory ELISA were the high number of FeLV-unexposed cats that tested antibody-positive (cohort 1—70/122, cohort 2—13/158) using a test cut-off of 16.3% compared to the positive control (pooled serum from experimentally FeLV-infected cats). It is not clear, therefore, to what degree the determination of anti-p15E antibodies is suitable for the determination of FeLV infection status. When the ELISA was first developed, serum samples from 294 cats in Switzerland were used to test the suitability of using the detection of anti-p15E antibodies for the diagnosis of FeLV infection. The sensitivity and specificity of p15E antibodies in experimentally infected cats were 95.7% and 100.0%, respectively. In naturally infected cats, the detection of anti-p15E antibodies showed a sensitivity of 77.1% and a specificity of 85.6% [49]. In this study, conditions of the experimentally infected cats had to be changed to reach an optimal trade-off between diagnostic sensitivity and specificity (for experimentally infected cats, a ROD cut-off of 0.0495 was used vs. 0.163 for naturally infected cats). Boenzli and colleagues also mentioned that the low specificity would probably have been much higher if the gold standard PCR assay used had been more sensitive and PCR results from organs in the privately owned cats in the present study had been available [49]. In contrast to experimentally infected cats, cats with a natural infection can have multiple organs affected, despite minor bone marrow involvement [16,46].

It is difficult to explain the detection of anti-p15E antibodies in a high number of FeLV-unexposed cats, particularly in the first cohort (Australian cats). One possibility is that endogenous FeLV plays a role. The presence of the transmembrane protein p15E has been described with the subtype FeLV-B [9]. Another possibility is that some of the cats categorized as FeLV-unexposed had been exposed to very low levels of FeLV; too low to cause NAb production, but high enough to be detectable by a sensitive laboratory p15E ELISA. This suggestion is supported by the findings in the FeLV-unexposed Australian cohort that older, male cats were more likely to have p15E laboratory ELISA-positive results than younger, female cats, possibly reflecting increased cumulative risk of low-level FeLV exposure through at-risk roaming behavior [37,74,75,76]. An Australian study reported FeLV infection or exposure in 13.2% (58/440) of cats tested compared to 7.5% (37/495) of cats tested in Munich, Germany [47,63], suggesting a higher level of FeLV exposure in Australia than Germany. In light of this, the cut-off value of the p15E laboratory ELISA, i.e., the point at which a sample is considered positive, should be critically reevaluated.

In addition, among FeLV-unexposed cats, it was observed that FIV-infected cats (19/19) and FIV-vaccinated cats (25/27) tested positive with the p15E laboratory ELISA. There might be a cross-reaction in the ELISA between p15E and FIV antibodies. This finding needs to be further investigated. In the meantime, especially in countries where vaccination against FIV is currently available (Australia, New Zealand, and Japan), or was previously available (North America), results from testing to detect the presence of anti-p15E antibodies should be interpreted with caution and should not be the sole method used to determine FeLV exposure or non-exposure. Instead, when FeLV infection or exposure is suspected, it is recommended to use other laboratory methods, such as FeLV proviral PCR testing, viral RT-PCR testing, and NAb testing [15].

Overall, v-RetroFel^®^FIV was able to accurately determine FIV infection status. Interestingly, the sensitivity of v-RetroFel^®^FIV testing in the present study was significantly higher in Australian cats than in German cats (94.7% vs. 30.0%). Another study reported reduced sensitivity (i.e., false-negative FIV results) in Swiss samples with PoC and laboratory ELISA testing, hypothesizing that the introduction of new FIV field isolates (e.g., due to increased travel) could have been responsible [66]. Similarly, it is possible that the seven FIV-infected cats in Germany that tested falsely negative with v-RetroFel^®^FIV were the result of genetic virus mutations and altered host antibody production. Little is known about genetic differences between FIV field isolates in Australia and Germany, and this could be an area for future research. It is also possible that the different criteria used to determine FIV infection status in Australian and German cats might have contributed to the different sensitivity rates reported. Due to the difference in test sensitivity between cohorts 1 and 2, v-RetroFel^®^FIV can be recommended for use particularly by veterinarians in Australia, with caution suggested if used in Germany.

With regards to v-RetroFel^®^FIV specificity, there was no significant difference when testing Australian cats compared to German cats (98.3% vs. 100%). This was despite the different testing criteria used, and 78 cats in cohort 2 had one band with WB and were categorized as FIV negative at the time of testing. Only p24 reacts in WB in the early acute phase of FIV infection before antibody development occurs, or in the end stage of FIV infection, due to immunodeficiency [66]. It is recommended to retest cats that are only p24-positive in the WB two to three months later [77]. However, there was 100% test agreement between WB and v-RetroFel^®^FIV in these 78 cases; therefore, discordant results due to early FIV infection did not appear to be a factor affecting test accuracy in the present study. Of particular interest to Australian vets will be the ability of the v-RetroFel^®^FIV test kit to differentiate FIV-vaccinated and FIV-infected cats, with all 27 uninfected FIV-vaccinated cats correctly testing negative (i.e., 100% specificity). v-RetroFel^®^FIV is the first p24/gp40 FIV kit reported in the scientific literature to be capable of DIVA, with three other FIV kits demonstrated to be capable of DIVA all detecting antibodies to gp40 only (Witness^®^, Anigen Rapid^®^ and RapidSTATUS™, Biotech Laboratories, Rockville, MD, USA) [60,78]. Currently, there is only one commercially available FIV vaccine (Fel-O-Vax^®^ FIV) sold in Australia, New Zealand, and Japan. It was also available in North America from 2003 to 2017, but has never been commercially available in Europe. Every jurisdiction should perform its own testing to determine the accuracy of any FIV PoC test kit, including v-RetroFel^®^FIV, prior to adopting them for use [61].

## 5. Conclusions

Measuring the antibody response to FeLV in cats with different FeLV outcomes and vaccination scenarios is complex and requires consideration of antibody response to both p15E and SU proteins. Currently, no single antibody test to determine the level of anti-p15E antibodies is completely reliable. FeLV antibody testing should always be carried out together with other laboratory tests, such as p27 antigen, proviral DNA PCR, and/or viral RNA testing, when trying to interpret antibody results. Furthermore, it should be remembered that the determination of FeLV infection status is always a snapshot and can change over time, for example, due to a weakening of the immune system. v-RetroFel^®^Ag/Ab (in its 2020–2021 version) did not reliably detect different FeLV infection outcomes and, therefore, does not currently offer any advantages over other available PoC tests that solely detect p27 antigen. Thus, its use cannot be recommended until improvements have been made. v-RetroFel^®^FIV was able to accurately determine FIV infection status, irrespective of a history of FIV vaccination, making it the first p24/gp40 FIV antibody PoC test kit reported to be capable of DIVA. However, of concern, and in need of additional investigation, was reduced test sensitivity in German cats.

## Figures and Tables

**Table 1:** Classification of feline leukemia virus (FeLV) infection status in Australian cats (*n* = 200). PoC = point-of-care, Ag = antigen, Lab = laboratory, NAb = neutralizing antibodies, NP = not performed.

FeLV Infection Status	Results			
	PoC p27 Ag	Lab-ELISA p27 Ag	Proviral DNA PCR	NAb
Progressive (*n* = 23)	+	+ ^1^	+	− ^2^
Regressive (*n* = 23)	−	− ^3^	+	+
Abortive (*n* = 32)	−	− ^4^	−	+
FeLV-unexposed (*n* = 122)	−	NP	−	−

^1^ Residual sample was available for laboratory-based p27 testing in 20/23 cats. ^2^ One cat was classified as progressively infected based on p27-antigenemia, but tested NAb-positive. ^3^ Residual samples were available for laboratory-based p27 testing in 20/23 cats. ^4^ Residual samples were available for laboratory-based p27 testing in 7/32 cats.

**Table 2:** Classification of feline leukemia virus (FeLV) infection status in German cats (*n* = 170). Lab = laboratory, Ag = antigen, RT = reverse-transcriptase, Ab = antibody, SU = surface unit protein.

FeLV Infection Status	Results				
	Lab-ELISA p27 Ag	Viral RT-PCR	Proviral DNA PCR	Lab-ELISA Anti-SU Ab	Lab-ELISA Anti-Whole Virus Ab
Progressive (*n* = 4)	+	+	+	−	−
Regressive (*n* = 2)	−	−	+	−	± ^2^
Abortive (*n* = 6)	−	−	−	+	+
FeLV-unexposed (*n* = 158)	−	−	−	± ^1^	± ^3^

^1^ Of the 158 FeLV-unexposed cats, 30 cats had anti-SU antibodies. Of these, five cats were vaccinated with a monovalent FeLV subunit p45 vaccine (Leucogen^®^, Virbac Animal Health) that is known to produce an antibody response. ^2^ One cat classified as regressively infected tested positive, and one cat classified as regressively infected tested negative, for anti-whole virus antibodies. ^3^ Of the 158 FeLV-unexposed cats, 11 cats had anti-whole virus antibodies, including two FeLV-vaccinated cats. One cat was vaccinated with a monovalent FeLV subunit vaccine (Leucogen^®^, Virbac Animal Health), and for one cat, the vaccine manufacturer was unknown.

**Table 3:** Results of v-RetroFel^®^Ag/Ab PoC testing to detect FeLV p27 antigen and FeLV p15E antibodies, and p15E laboratory ELISA testing to detect p15E antibodies, in Australian cats (*n* = 200). Positive results are shown. Refer to Table 1 for the testing approach used to classify cats in Australia as progressively infected, regressively infected, abortively infected, or FeLV-unexposed. p15E antibody test results were not used for classification of FeLV infection status. None of the progressively infected or regressively infected cats had been vaccinated against FeLV, 25/32 abortively infected cats had been vaccinated against FeLV (11 with Fel-O-Vax^®^ Lv-K and 14 with Fel-O-Vax^®^ 5), and 82/122 FeLV-unexposed cats had been vaccinated against FeLV (27 with Fel-O-Vax^®^ Lv-K, 36 with Fel-O-Vax^®^ 5, and 19 with Leucogen^®^). FeLV = feline leukemia virus, PoC = point-of-care, Ag = antigen, Ab = antibody, Lab = laboratory.

FeLV Infection Status	Positive Results		
	v-RetroFel^®^Ag PoC p27 Ag	v-RetroFel^®^Ab PoC p15E Ab	Lab-ELISA p15E Ab
Progressive	21 (91%)	13 (57%)	16 (70%)
(*n* = 23)			
Regressive	0 (0%)	0 (0%)	16 (70%)
(*n* = 23)			
Abortive	0 (0%)	0 (0%)	25 (78%)
(*n* = 32)			
FeLV-unexposed	1 (0.8%)	12 (10%) ^1^	70 (57%)
(*n* = 122)			

^1^ Only 2/12 of these cats also tested p15E antibody-positive with the laboratory ELISA. All 12 cats that were p15E antibody-positive with v-RetroFel^®^Ab PoC testing had been vaccinated against FeLV (5 cats with Fel-O-Vax^®^ Lv-K, 4 cats with Fel-O-Vax^®^ 5, and 3 cats with Leucogen^®^).

**Table 4:** Results of v-RetroFel^®^FIV PoC testing to detect antibodies against FIV capsid protein (p24) and glycoprotein (gp40) in 200 Australian cats (cohort 1). FIV infection status was determined by results from three commercially available PoC tests, PCR testing to confirm FIV-positive results, and virus isolation to confirm FIV infection in two FIV-vaccinated cats. FIV = feline immunodeficiency virus, PoC = point-of-care.

FIV Infection Status	v-RetroFel^®^FIV PoC Result	
	Negative	Positive
Uninfected	178	3
(*n* = 181)		
Infected	1	18
(*n* = 19)		

**Table 5:** Results of v-RetroFel^®^Ag/Ab PoC testing to detect FeLV p27 antigen and FeLV p15E antibodies, and p15E laboratory ELISA testing to detect p15E antibodies, in German cats (*n* = 170). Positive results are shown. Refer to Table 2 for the testing approach used to classify cats in Germany as progressively infected, regressively infected, abortively infected, or FeLV-unexposed. p15E antibody test results were not used for classification of FeLV infection status. None of the progressively infected, regressively infected, or abortively infected cats had been vaccinated against FeLV, and 11/158 FeLV-unexposed cats had been vaccinated against FeLV (5 cats with Purevax^®^, 4 cats with Leucogen^®^, and 2 cats with an unknown vaccine). FeLV = feline leukemia virus, PoC = point-of-care, Ag = antigen, Ab = antibody, Lab = laboratory.

FeLV Infection Status	Positive Results		
	v-RetroFel^®^Ag PoC p27 Ag	v-RetroFel^®^Ab PoC p15E Ab	Lab-ELISA p15E Ab
Progressive	4 (100%)	0 (0%)	3 (75%)
(*n* = 4)			
Regressive	0 (0%)	0 (0%)	0 (0%)
(*n* = 2)			
Abortive	0 (0%)	1 (17%) ^1^	2 (33%)
(*n* = 6)			
FeLV-unexposed	0 (0%)	10 (6%) ^2^	13 (8%) ^3^
(*n* = 158)			

^1^ The one abortive infection that tested positive with the v-RetroFel^®^Ab PoC test was negative with the p15E laboratory ELISA. ^2^ One of these ten cats also tested positive with the p15E laboratory ELISA. Two FeLV-vaccinated cats tested positive with v-RetroFel^®^Ab and negative with the p15E laboratory ELISA (one cat was vaccinated with Purevax^®^ FeLV shortly before sampling, and one cat had been vaccinated eight times with Leucogen^®^). ^3^ Three of these 13 cats had been vaccinated against FeLV (one cat with Purevax^®^ FeLV; one cat with Leucogen^®^; and for one cat, the vaccine manufacturer was unknown).

**Table 6:** Results of v-RetroFel^®^FIV PoC testing to detect antibodies against FIV capsid protein (p24) and glycoprotein (gp40) in 170 German cats (cohort 2). FIV infection status was determined by results from Western blotting (WB). FIV = feline immunodeficiency virus, PoC = point-of-care.

FIV Infection Status	v-RetroFel^®^FIV PoC Result	
	Negative	Positive
Uninfected ^1^	160	0
(*n* = 160)		
Infected	7	3
(*n* = 10)		

**^1^** Seventy-eight cats had one band with WB and were categorized as FIV-negative at the time of testing. The other 82 cats had no bands with WB.

## Data Availability

All data presented in this paper are available upon request.
